# Leguminous green manure enhances soil quality and plant productivity in coal mine reclaimed lands: a decade-long field study

**DOI:** 10.3389/fpls.2026.1802650

**Published:** 2026-06-17

**Authors:** Ying Dong, Yi-Jie Quan, Huo-Feng Zhang, Yan Yang, Dong-He Xue, Hui-Juan Bo, Jiang-Hong Bo, Bian-Hua Zhang, Dong-Sheng Jin, Wen-Jing Zhang, Qiang Zhang, Ming-Gang Xu, Wei Wang

**Affiliations:** 1 Engineering Technology Innovation Center for Ecological Protection and Restoration in the Middle Yellow River, Ministry of Natural Resources, Taiyuan, China; 2 Department of Resources and Environment, Shanxi Agricultural University, Taiyuan, China; 3 Soil Health Laboratory in Shanxi Province, Taiyuan, China; 4 Department of Geography, Xinzhou Teachers University, Xinzhou, China

**Keywords:** green manure legume crops, lignin content, natural restoration strategy, reclaimed soil, soil physicochemical and biochemical properties

## Abstract

Green manure planting is a commonly used environmentally friendly and sustainable field management practice in resource-constrained agricultural ecosystems. However, the effects of green manure on soil quality and plant production in coal mine reclamation agroecosystems remain unclear. In this study, a decade-long legume cultivation experiment was conducted to evaluate the effects on soil aggregate structure, nutrients, microbial diversity, and plant productivity. The experiment was established at the Gujiao long-term monitoring site of Shanxi Agricultural University and included three treatments: natural restoration, alfalfa planting and villose vetch planting. Compared with the control, legume planting—particularly alfalfa—significantly increased plant biomass and the accumulation of soil organic carbon (SOC) and total phosphorus. Specifically, in the 0–20 cm soil layer under the RAF treatment, SOC and TP contents increased by 23.84% and 47.17%, respectively. Moreover, alfalfa planting enhanced the proportion of aggregates larger than 0.25 mm by 36.56% in wet sieving. Interestingly, continuous legume forage cultivation significantly improved soil aggregate stability. Moreover, alfalfa planting increased bacterial diversity, stimulated the accumulation of lignin-derived compounds, and resulted in the highest acid–formaldehyde ratio, indicating that alfalfa planting increased lignin degradation capacity and provided beneficial nutrients for reclaimed soil. Variance partitioning analysis indicated that both soil physicochemical properties and the microbial community structure co-driven plant biomass variations. These findings clarify the mechanisms by which legume cultivation improves soil quality, enhances microbial diversity, and increases biomass production. In conclusion, this study supports the application of leguminous green manure as a natural solution for coal mine land reclamation, contributing to soil quality improvement and fostering sustainable development in postmining ecosystems.

## 1 Introduction

With the acceleration of industrialization, the exploitation of mineral resources has posed significant challenges to the natural ecological environment, despite serving as a major driver of socioeconomic development (Shao et al., 2023). As a critical component of ecological restoration, mine reclamation has gained increasing importance (Ren et al., 2025). Mine reclamation not only is essential for the sustainable utilization of land resources but also serves as a key strategy for maintaining regional ecological balance, conserving biodiversity, and improving the living conditions of local communities (Guan et al., 2022). Among various reclamation strategies, the restoration and enhancement of soil quality lie at the core, as they directly determine whether the reclaimed land can regain its ecological functionality and productive potential (Ren et al., 2023).

The use of green manure crops has garnered considerable attention for its role in improving soil quality and agricultural productivity (Wei et al., 2025a). Among these crops, legumes stand out due to their unique biological nitrogen fixation capabilities, demonstrating substantial potential for enhancing soil health (Akchaya et al., 2025). Through a symbiotic relationship with rhizobacteria, legumes convert atmospheric nitrogen into plant-available forms, thereby providing a continuous and sustainable nitrogen supply to the soil (Barbieri et al., 2023). This natural nitrogen fixation mechanism not only reduces the reliance on synthetic nitrogen fertilizers but also mitigates the environmental risks associated with fertilizer overuse (Huang et al., 2025). Moreover, legume growth enhances soil aeration, water infiltration, and moisture retention while promoting soil aggregate formation (Li et al., 2025a; Ma et al., 2025; Stainsby et al., 2020). These improvements create a conducive environment for soil microbial communities, facilitating their activity and supporting a positive feedback loop that sustains overall soil ecosystem function (Kanté et al., 2021). Additionally, the biomass of legumes and their lignin content are critical factors influencing soil quality (Griffin and Jungers, 2025). Legumes produce substantial biomass, and the decomposition of both root and above-ground residues supplies considerable amounts of organic matter to the soil. The presence of lignin facilitates the stable accumulation of this organic matter and promotes humus formation, thereby further enhancing soil fertility and structural stability (Gao et al., 2025).

In recent years, the critical role of soil microorganisms in ecosystem functioning has received increasing attention (Bianchi et al., 2024). Soil microbial communities, including bacteria and fungi, are essential components of soil ecosystems and actively participate in the decomposition of organic matter, nutrient cycling, and the formation and stabilization of the soil structure (Yasen et al., 2025). The microbial diversity index serves as a key indicator of soil microbial community health and functionality, with high diversity generally reflecting increased ecosystem stability and enhanced ecological functions (Hao et al., 2024). Legume cultivation can markedly alter the structure and function of soil microbial communities by increasing organic matter inputs and improving soil environmental conditions, thereby exerting significant effects on soil quality (Lira Junior et al., 2020; Wang et al., 2025). However, the specific mechanisms by which legume green manure improves the quality of reclaimed soils in coal mine areas remain insufficiently understood. Based on the above information, we hypothesized that the cultivation of leguminous green manure crops enhances soil organic carbon and plant biomass by improving the soil structure, increasing the soil lignin and nutrient contents, and optimizing the microbial community composition. To test this hypothesis, a long-term field experiment spanning ten consecutive years was conducted to elucidate the mechanisms by which different leguminous green manure crops influence soil aggregate stability, nutrient status, lignin accumulation, and biomass production. The main objectives of this study were to (1) investigate the effects of different green manure legume crops on soil aggregate structure in reclaimed coal mine soils; (2) assess the impact of legume cultivation on lignin content in these soils; (3) evaluate changes in bacterial and fungal diversity in reclaimed soils in response to green manure legume treatments; and (4) determine the interactions between microbial communities and nutrient dynamics under legume cultivation and their influence on plant biomass. This study aims to provide novel insights into improving the quality and functional efficiency of reclaimed soils in mining areas and support the application of nature-based restoration strategies focused on green manure crop cultivation.

## 2 Materials and methods

### 2.1 Experimental site

**Figure 1:**
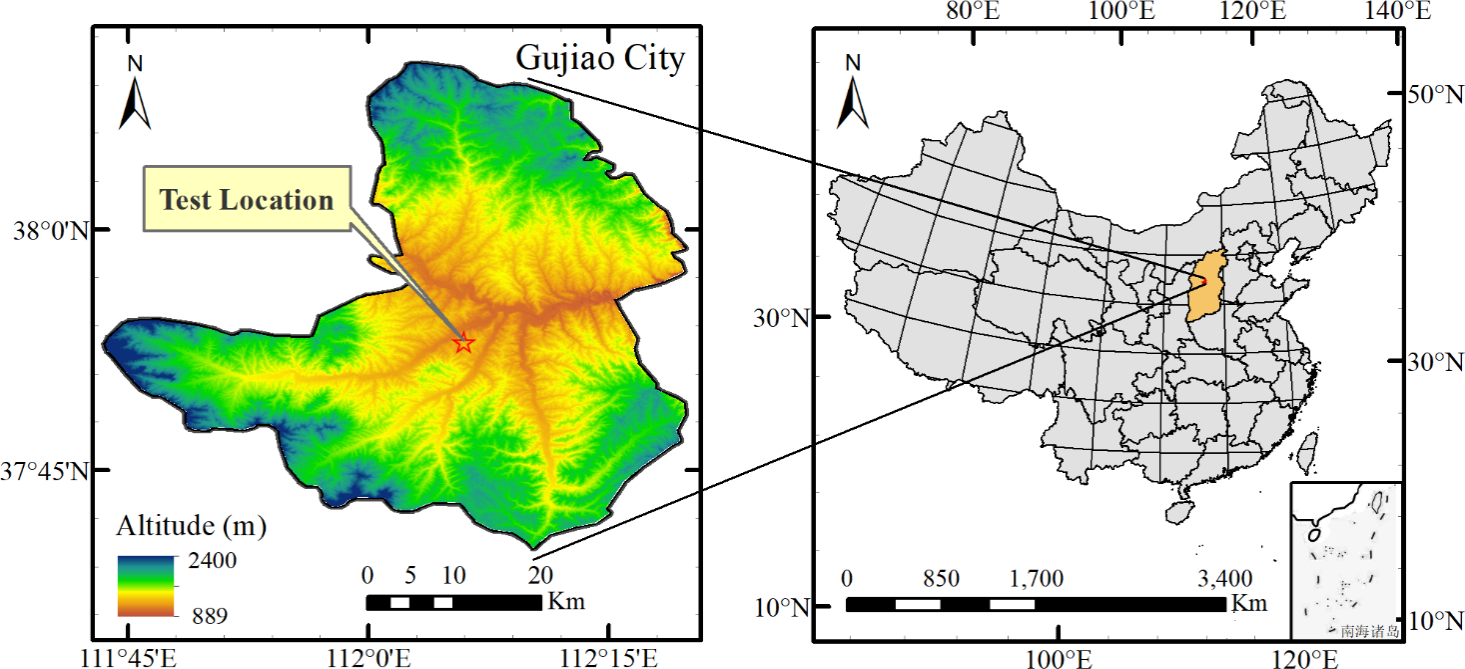
Map showing the location of the study area.

The study was conducted at the long-term positioning experimental base in Gujiao, Soil Health Laboratory, Shanxi Agricultural University (37°53′ N, 112°06′ E). The site is characterized by a northern temperate continental climate, featuring pronounced diurnal temperature variation, an average annual precipitation of approximately 460 mm, and evapotranspiration of approximately 1025 mm, indicative of distinct arid conditions (**Figure 1**).

### 2.2 Experimental design

This long-term positioning experiment was initiated in 2013 and employed a randomized block design with three treatments: natural restoration (control, CK), alfalfa planting (RAF), and villose vetch (VVR) planting, each replicated three times. Each plot measured 15 m × 6 m (90 m²). Both alfalfa and villose vetch were sown in strips and furrows at a controlled depth of 3 cm, with the soil ploughed prior to sowing. In contrast, the control plots were maintained in their original state without ploughing. A compound fertilizer (N:P:K = 28:12:10) was uniformly applied to all the plots at a rate of 600 kg ha⁻¹, followed by thorough incorporation using a rotary tiller to ensure even nutrient distribution.

### 2.3 Sample collection

The soil samples used in this study were collected in October 2023. Within each plot, five sampling points were combined into a composite sample following a five-point sampling method. Fresh soil samples were sieved through 2 mm mesh to remove roots and debris. The processed samples were then divided into two portions: one was air-dried for physicochemical analyses, while the other was transported to the laboratory under low-temperature conditions in sealed containers for microbial diversity assessment. The soil aggregate samples of 0–20 cm and 20–40 cm were collected with PVC pipe (diameter is 10 cm, height is 20 cm). For each soil aggregate samples, a five-point sampling method is also used, in which samples from the same treatment and the same soil layer are mixed together. After sampling, its were divided into diameter 1 cm small clods for air-drying.

### 2.4 Measurement indices

In this study, soil aggregate particle size was determined using both dry and wet sieving methods. The aggregates were classified into five size fractions: >5.00 mm, 2.00–5.00 mm, 0.25–2.00 mm, 0.05–0.25 mm, and<0.05 mm (Zhao et al., 2024). Based on the particle size distribution data, the mean weight diameter (MWD) of the soil aggregates was calculated following the method described by Wu et al. (2024) (Equation 1). The calculation formula is as follows:

(1) $$\text{MWD}=\frac{\sum \left(d_{i}\times w_{i}\right)}{\sum w_{i}}$$

where di denotes the mean diameter of the ith particle size classification and wi is the proportion of that particle size classification to the total sample weight.

Soil organic carbon content was determined using the potassium dichromate oxidation method with external heating (Mao et al., 2024). Soil total nitrogen (TN) was measured via Kjeldahl digestion followed by fully automated nitrogen determination (Bao, 2000). Total phosphorus (TP) content was quantified using the molybdenum-antimony colorimetric method (Bao, 2000). Microbial diversity was assessed through 16S rRNA gene sequencing. PCR amplification of bacteria was performed using 338F’ (ACTCCTACGGGAGGCAGCA) and 806R (GGACTACHVGTWTCTAAT), whereas that of fungi was performed using ITS5 (GGAAGTAAAAGTCGTAACAAGG) and ITS2 (GCTGCGTTCTTCATCGATGC) (Ahsan et al., 2024). Lignin phenolic compounds were analysed by copper oxide oxidation (Ma et al., 2018) and quantified using gas chromatography–mass spectrometry (GC–MS; Agilent 7890B GC coupled with Agilent 5977B MSD, California, USA). The analysis included the quantification of para-hydroxy lignans (H), vanillin-based phenolic (V), lilac-based phenolic (S), and cinnamon-based phenolic (C) compounds, as well as the acid–formaldehyde ratios of vanillin-based phenolic (V) and lilac-based phenolic (S) compounds (Abiven et al., 2011; Ma et al., 2023). Plant biomass was determined by harvesting vegetation within a 1 m × 1 m subplot in each plot, and biomass values were calculated accordingly.

### 2.5 Statistical analysis

One-way analysis of variance (ANOVA) was performed using SPSSAU (https://spssau.com) to evaluate the effects of different land use practices on soil physicochemical properties, aggregate particle size distributions, lignin contents, and microbial diversity indices (Ma et al., 2023). Redundancy analysis (RDA) was conducted with CANOCO 5 to investigate the relationships between the microbial diversity indices and the soil physicochemical parameters. Pearson correlation analysis was used to assess correlations among variables, with results visualized via heatmaps (Wu et al., 2024). Variance partitioning analysis (VPA) was carried out in R to quantify the contributions of factors strongly correlated with biomass variation. Graphical representations were generated using Origin 2024, R version 4.4.1, and Canoco 5.

## 3 Results

### 3.1 Biomass

**Figure 2:**
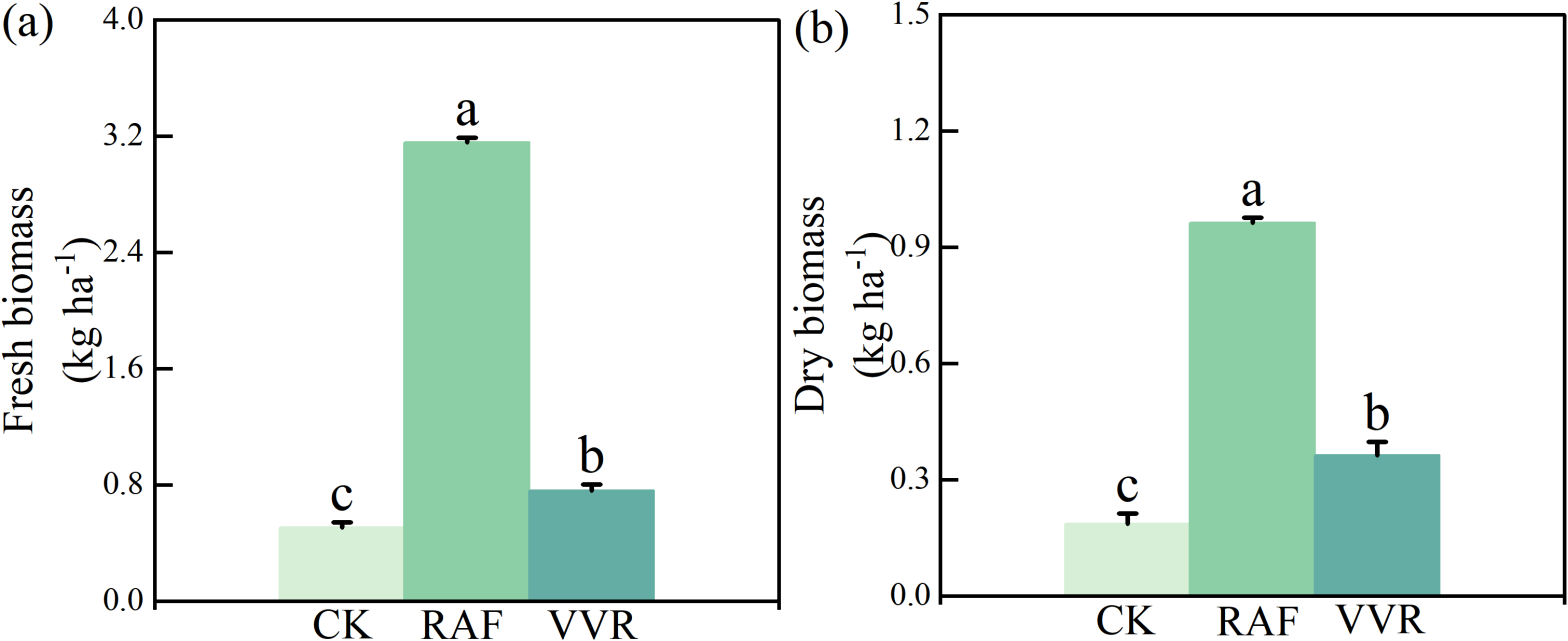
Changes in plant fresh biomass **(a)** and dry biomass **(b)** at different treatment levels. CK, natural restoration site. RAF, 10 years of continuous alfalfa planting on reclaimed land. VVR, 10 years of continuous planting of hairy camphor on reclaimed land. Different lowercase letters represent the differences between treatments reaching significance at the 0.05 level (n=3). The same notation applies to subsequent figures.

Aboveground biomass differed significantly among the three treatments (**Figure 2**). Compared with the control (CK), both the RAF and VVR treatments markedly increased fresh weight accumulation, with the RAF treatment resulting in the greatest increase in fresh weight accumulation. The dry weight showed the same trend as the fresh weight, with both the RAF and VVR treatments having significantly greater values than the CK, and the RAF treatment significantly outperformed the VVR treatment.

### 3.2 Soil physicochemical properties

Leguminous pastures established through green manure cultivation significantly increased the soil nutrient contents (**Table 1**). In the 0–20 cm soil layer, the soil organic carbon (SOC) and total phosphorus (TP) contents increased by approximately 23.84% and 47.17%, respectively, in the RAF treatment group compared with those in the control group, whereas the carbon-to-nitrogen (C:N) ratio increased by approximately 6.93%; for the VVR treatment, these parameters were elevated by 7.58%, 6.85%, and 3.91%, respectively, compared to those in the control. In the 20–40 cm soil layer, the RAF treatment resulted in increases of 25.41% in SOC, 5.19% in TP, and 11.09% in the C:N ratio relative to those in the control. TN content was highest under the VVR treatment, increased by 2.94% compared with that of the control.

**Table 1:** Physicochemical properties of 0–40 cm soil depth under three treatments.

Soil depth (cm)	Tre.	SOC (g kg^-1^)	TN (g kg^-1^)	TP (g kg^-1^)	C:N	N:P	C:P
0-20	CK	8.60 ± 0.34b	0.54 ± 0.01c	0.53 ± 0.02c	14.56 ± 2.09a	0.44 ± 0.05b	6.42 ± 0.90a
	RAF	10.65 ± 0.38a	0.60 ± 0.01b	0.78 ± 0.02a	15.57 ± 1.51a	0.49 ± 0.03a	7.66 ± 0.75a
	VVR	9.90 ± 0.35a	0.65 ± 0.05a	0.73 ± 0.02b	15.92 ± 0.52a	0.49 ± 0.07a	7.87 ± 0.34a
20-40	CK	4.92 ± 0.69b	0.34 ± 0.03b	0.77 ± 0.01b	15.74 ± 0.60b	1.04 ± 0.03a	16.38 ± 0.59a
	RAF	6.17 ± 0.59a	0.40 ± 0.04a	0.81 ± 0.04a	17.49 ± 0.66a	0.77 ± 0.04c	13.50 ± 0.44b
	VVR	5.24 ± 0.25ab	0.33 ± 0.05c	0.67 ± 0.02c	15.24 ± 0.49b	0.88 ± 0.07b	13.48 ± 0.44b

CK, control group. RAF, alfalfa. VVR, vicia villosa roth. Lowercase letters indicate significant differences at 0.05 level among same soil layers. Values represent means ± standard errors. The same as below.

### 3.3 Distribution and stability of soil aggregates

The continuous cultivation of leguminous green manure significantly improved the soil aggregation structure (**Tables 2**, **3**). Compared with the control, among the dry sieving treatments (**Table 2**), the RAF treatment increased the proportion of aggregates larger than 0.25 mm in the 0–20 cm soil layer by approximately 7.27% and the MWD by approximately 4.84%, whereas the VVR treatment resulted in the highest proportion of >0.25 mm aggregates, increasing by approximately 8.62% relative to that in the control. Both treatments elevated the proportion of 0.25–5.00 mm aggregates and enhanced the distribution of the aggregate structure. In the 20–40 cm soil layer, the proportion of >0.25 mm aggregates and MWD increased by approximately 33.74% and 23.26%, respectively, in the RAF treatment compared with the control, and the proportions of aggregates in the 2.00–5.00 mm and >5.00 mm size classes increased by approximately 22.57% and 72.41%, respectively.

**Table 2:** Analysis of dry sieve particle size of 0-40cm soil aggregates under three treatments.

Soil depth (cm)	Tre.	Distribution of the five sizes (%)					> 0.25	MWD
		< 0.05	0.05-0.25	0.25-2.00	2.00-5.00	> 5.00		
0-20	CK	4.39 ± 1.09a	27.33 ± 1.76a	13.67 ± 2.15b	13.74 ± 2.04a	40.82 ± 3.72a	68.24 ± 2.84b	46.86 ± 1.21b
	RAF	5.06 ± 1.00a	21.75 ± 2.92b	18.10 ± 3.00a	12.47 ± 1.72a	42.63 ± 6.78a	73.20 ± 3.57a	49.13 ± 1.79ab
	VVR	5.12 ± 1.36a	20.76 ± 0.97b	18.26 ± 1.96a	13.90 ± 1.87a	41.96 ± 4.40a	74.12 ± 8.06a	49.57 ± 0.80a
20-40	CK	5.18 ± 0.74a	34.50 ± 2.20a	14.65 ± 1.20a	14.80 ± 2.94b	30.87 ± 4.90b	60.32 ± 2.47b	43.00 ± 1.44b
	RAF	2.42 ± 0.68b	16.93 ± 1.20b	12.07 ± 0.80b	18.16 ± 3.43a	53.22 ± 5.39a	80.65 ± 1.52a	53.01 ± 0.68a
	VVR	5.90 ± 0.27a	32.57 ± 4.02a	16.01 ± 1.94a	13.90 ± 0.80b	31.62 ± 5.21b	61.53 ± 3.95b	43.49 ± 1.87b

**Table 3:** Analysis of wet sieve particle size of 0-40cm soil aggregates under three treatments.

Soil depth (cm)	Tre.	Distribution of the five sizes (%)					>0.25	MWD
		<0.05	0.05-0.25	0.25-2.00	2.00-5.00	>5		
0-20	CK	10.05 ± 1.44b	31.88 ± 1.93a	17.25 ± 2.60c	13.08 ± 1.66a	27.75 ± 2.03a	58.08 ± 2.09b	41.32 ± 1.09b
	RAF	9.77 ± 2.41b	10.95 ± 1.58b	51.19 ± 6.07b	9.73 ± 5.16a	18.47 ± 3.73b	79.28 ± 2.91a	51.44 ± 1.64a
	VVR	14.59 ± 2.08a	5.97 ± 2.69c	74.68 ± 2.24a	3.60 ± 1.11b	2.17 ± 0.49c	80.45 ± 2.19a	51.54 ± 1.52a
20-40	CK	11.98 ± 0.81b	27.84 ± 6.13a	50.20 ± 8.79b	4.60 ± 1.39a	5.40 ± 2.59a	60.19 ± 6.48b	42.09 ± 6.48b
	RAF	8.68 ± 1.50c	6.13 ± 5.85b	80.86 ± 9.59a	3.35 ± 3.05a	0.99 ± 0.99b	85.20 ± 6.76a	54.38 ± 6.76a
	VVR	15.15 ± 2.72a	22.97 ± 2.05a	57.62 ± 2.56b	3.08 ± 0.86a	1.20 ± 8.07b	61.89 ± 3.50b	42.50 ± 3.49b

Among the wet sieving treatments (**Table 3**), the RAF treatment increased the proportion of aggregates larger than 0.25 mm in the 0–20 cm soil layer by 36.56% compared with that in the control, and the proportion of aggregates 0.25–2.00 mm in size increased by 196.46%. The VVR treatment presented the highest proportion within the 0.25–2.00 size class, with an increase of 332.35%. Both green manure treatments not only increased the proportion of aggregates but also resulted in higher MWD values than those in the control, with increases of 24.49% and 24.73% under the RAF and VVR treatments, respectively. In the 20–40 cm soil layer, the RAF treatment increased the proportion of >0.25 mm aggregates and the MWD by 41.55% and 29.21%, respectively, compared with those in the control, representing the highest values among the three treatments. The proportion of aggregates in the 0.25–2.00 mm size class was 80.86% under RAF, which was 23.24% greater than that observed under the VVR treatment.

### 3.4 Microbial diversity indices

According to the microbial diversity analysis of the 0–20 cm soil layer, the legume pasture treatments significantly increased the bacterial community diversity (**Table 4**). The Shannon index of bacteria was approximately 0.45% higher under the RAF treatment than under the control treatment. In contrast, the VVR treatment resulted in an increase in bacterial abundance, as indicated by a 4.40% increase in the Chao1 index relative to that of the control. With respect to the fungal community, the RAF treatment resulted in a slightly lower Shannon diversity index than that observed for the control, with a decrease of approximately 7.90%; however, it still maintained relatively high fungal diversity. The VVR treatment resulted in reduced fungal richness, with the Chao1 index decreasing by approximately 13.10% compared with that of the control; however, the VVR treatment resulted in an increase in fungal diversity, with a Shannon index approximately 5.10% greater than that of the control.

**Table 4:** Bacterial and Fungus diversity index of 0–20 cm soil depth under three treatments.

Microbe	Tre.	Chao1	Goods_coverage	Observed_species	Pielou_e	Shannon	Simpson
Bacteria	CK	4176.14 ± 130.54b	0.998 ± 0.001a	4147.34 ± 108.53a	0.920 ± 0.00b	11.05 ± 0.44a	0.9990 ± 0.00b
	RAF	4241.63 ± 274.90a	0.996 ± 0.001b	4170.62 ± 225.18a	0.923 ± 0.00a	11.10 ± 0.86a	0.9991 ± 0.00a
	VVR	4358.16 ± 260.08a	0.996 ± 0.012b	4254.76 ± 185.62a	0.920 ± 0.00b	11.08 ± 0.77a	0.9991 ± 0.00a
Fungus	CK	468.68 ± 11.87a	0.9992 ± 0.00b	452.33 ± 9.74a	0.701 ± 0.05a	6.18 ± 0.49a	0.918 ± 0.05a
	RAF	397.86 ± 11.20b	0.9993 ± 0.00b	385.10 ± 13.23b	0.623 ± 0.02a	5.35 ± 0.20a	0.898 ± 0.00a
	VVR	398.55 ± 2.02b	0.9997 ± 0.00a	396.27 ± 2.80b	0.671 ± 0.01a	5.79 ± 0.82a	0.945 ± 0.01a

### 3.5 Heatmap analysis of the correlation between lignin content and physicochemical and biological properties

**Figure 3:**
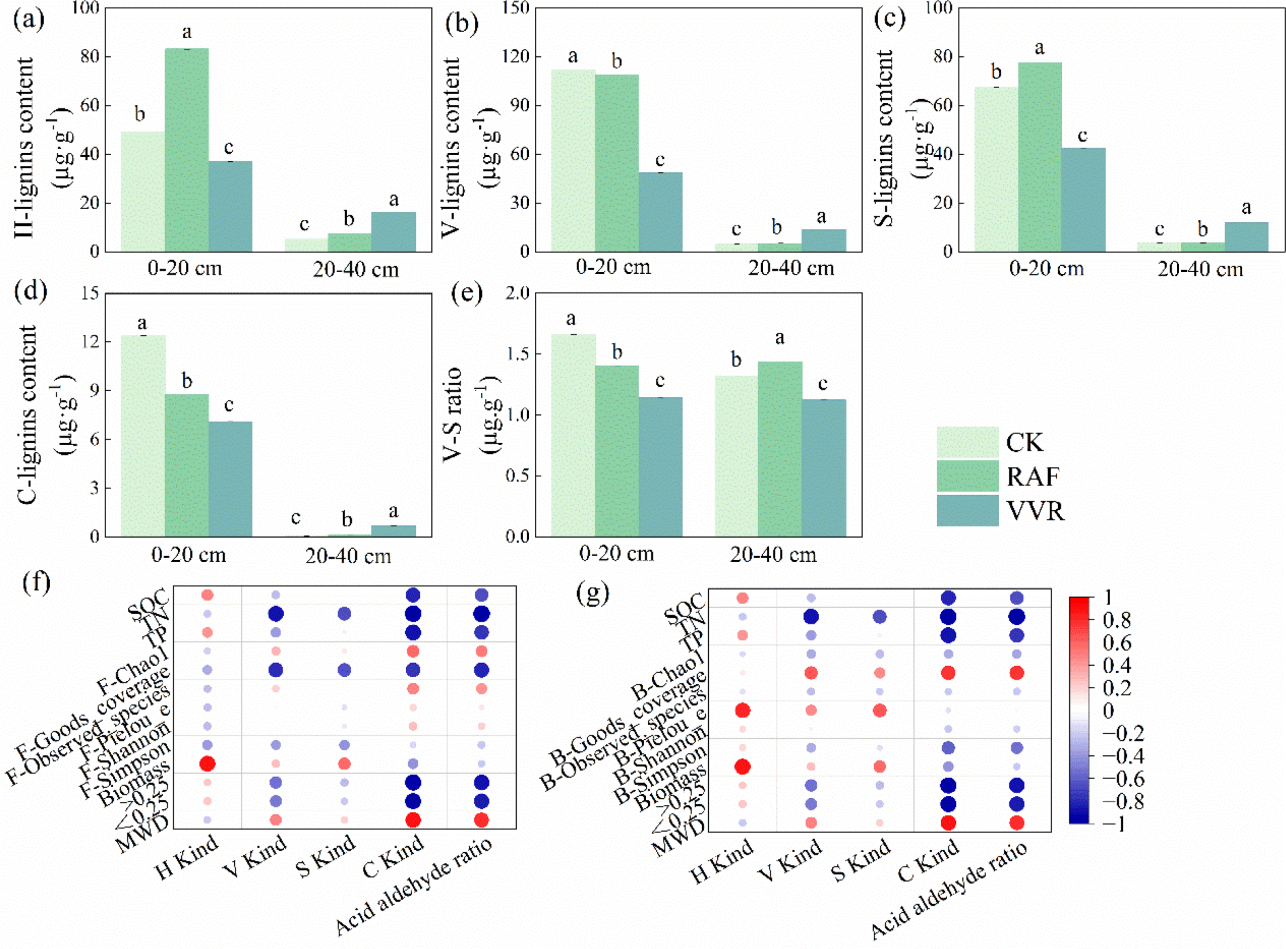
The variations of soil lignin contents in response to the three treatments **(a-e)** and heat map correlation analysis at different treatment levels **(f, g)**. **(a)** Para-hydroxy lignans content. **(b)** Vanilla-based phenolic content. **(c)** Lilac-based phenolic content. **(d)** Cinnamon-based phenolic content. **(e)** Acid-formaldehyde ratio. **(f)** Soil lignin content correlation analyses with soil aggregate and bacterial community diversity. **(g)** Soil lignin content correlation analyses with soil aggregate and fungal community diversity.

Overall, the soil lignin content significantly decreased with increasing soil depth (**Figures 3a–e**). In the 0–20 cm soil layer, the lignin content under the RAF treatment was significantly greater than that under the other treatments, particularly for the H-, V-, and S-type compounds. Although lignin fractions decreased in the 20–40 cm soil layer, the relative trends among the treatments remained consistent. The acid–formaldehyde ratios in the topsoil were greatest in the RAF and CK treatments, whereas in the deeper soil layer, this ratio was greater in the VVR treatment than in the CK.

Correlation heatmap analysis revealed that within the bacterial-dominated community (**Figure 3f**), the lignin H fraction was significantly positively correlated with plant biomass, soil organic carbon, and total phosphorus. In contrast, the lignin V and S fractions were significantly negatively correlated with soil total nitrogen and the bacterial Good’s coverage index. The lignin C fractions and acid–to-formaldehyde ratios were significantly positively correlated with the mean aggregate diameter but significantly negatively correlated with soil organic carbon, total nitrogen, and total phosphorus.

In the fungal-dominated community analysis (**Figure 3g**), the lignin H fraction was significantly positively correlated with plant biomass and the fungal Pielou’s evenness index. The lignin V and S fractions were significantly negatively correlated with soil total nitrogen. Similarly, the lignin C fractions and acid–formaldehyde ratios were significantly positively correlated with the mean soil aggregate diameter and Good’s coverage index but negatively correlated with the soil organic carbon, total nitrogen, and total phosphorus contents.

### 3.6 Redundancy analysis of soil physicochemical properties and microbial diversity indices

**Figure 4:**
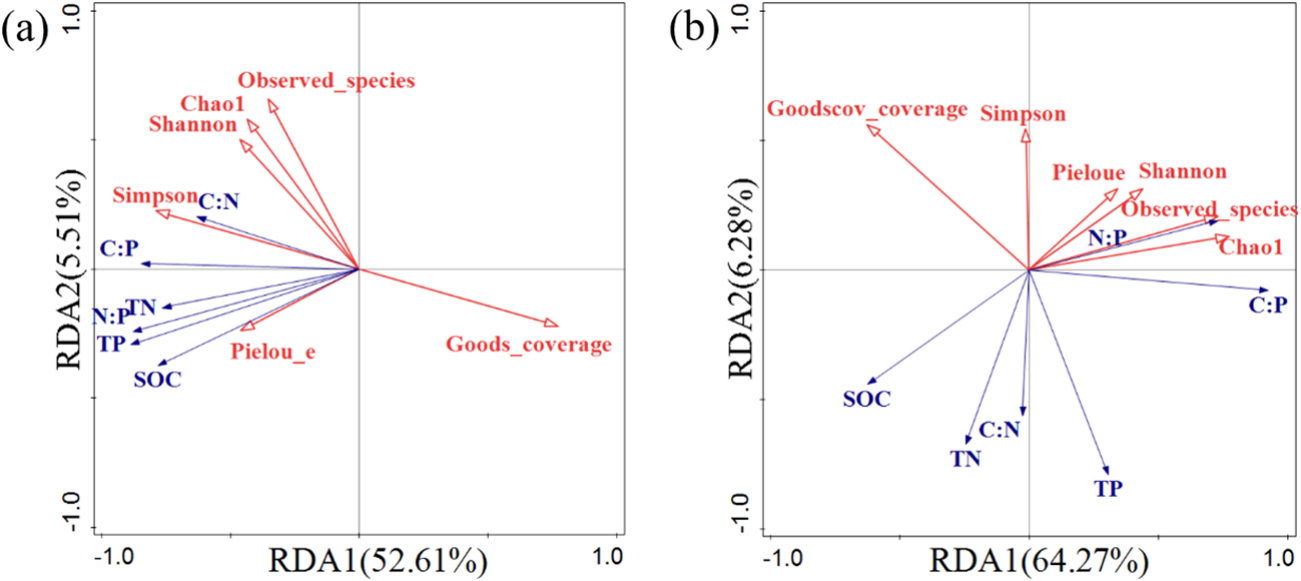
Redundancy analysis of soil microbial community diversity and soil physico-chemical properties. **(a)** Redundancy analysis of soil bacterial diversity index with soil physico-chemical. **(b)** Redundancy analysis of soil fungal community with physico-chemical properties. SOC, soil organic carbon; TN, soil total nitrogen; TP, total phosphorus; C:N, the ratio of soil organic carbon to total nitrogen; C:P, the ratio of soil organic carbon to total phosphorus; N:P, the ratio of soil total natrogen to total phosphorus.

Redundancy analysis (RDA) revealed significant correlations between the bacterial community composition and the soil physicochemical properties as well as the microbial diversity indices (**Figure 4a**). On the RDA1 axis, soil organic carbon and total nitrogen were strongly associated with bacterial diversity metrics, including the Chao1 and Shannon indices. The carbon-to-nitrogen (C:N) ratio also exhibited a significant relationship with bacterial community distribution, highlighting the crucial influence of soil nutrient status on bacterial community structure.

For the fungal community (**Figure 4b**), redundancy analysis (RDA) revealed significant correlations between the soil nitrogen-to-phosphorus (N:P) ratios and several diversity indices, including the Shannon, Chao1, and Observed species indices. These findings suggest that soil nutrient chemistry plays a key role in shaping fungal community structure. Additionally, Pielou’s evenness index was closely associated with the N:P ratio distribution, further underscoring the influence of both soil organic matter and microbial diversity on fungal community composition.

### 3.7 Correlation heatmaps and VPA

**Figure 5:**
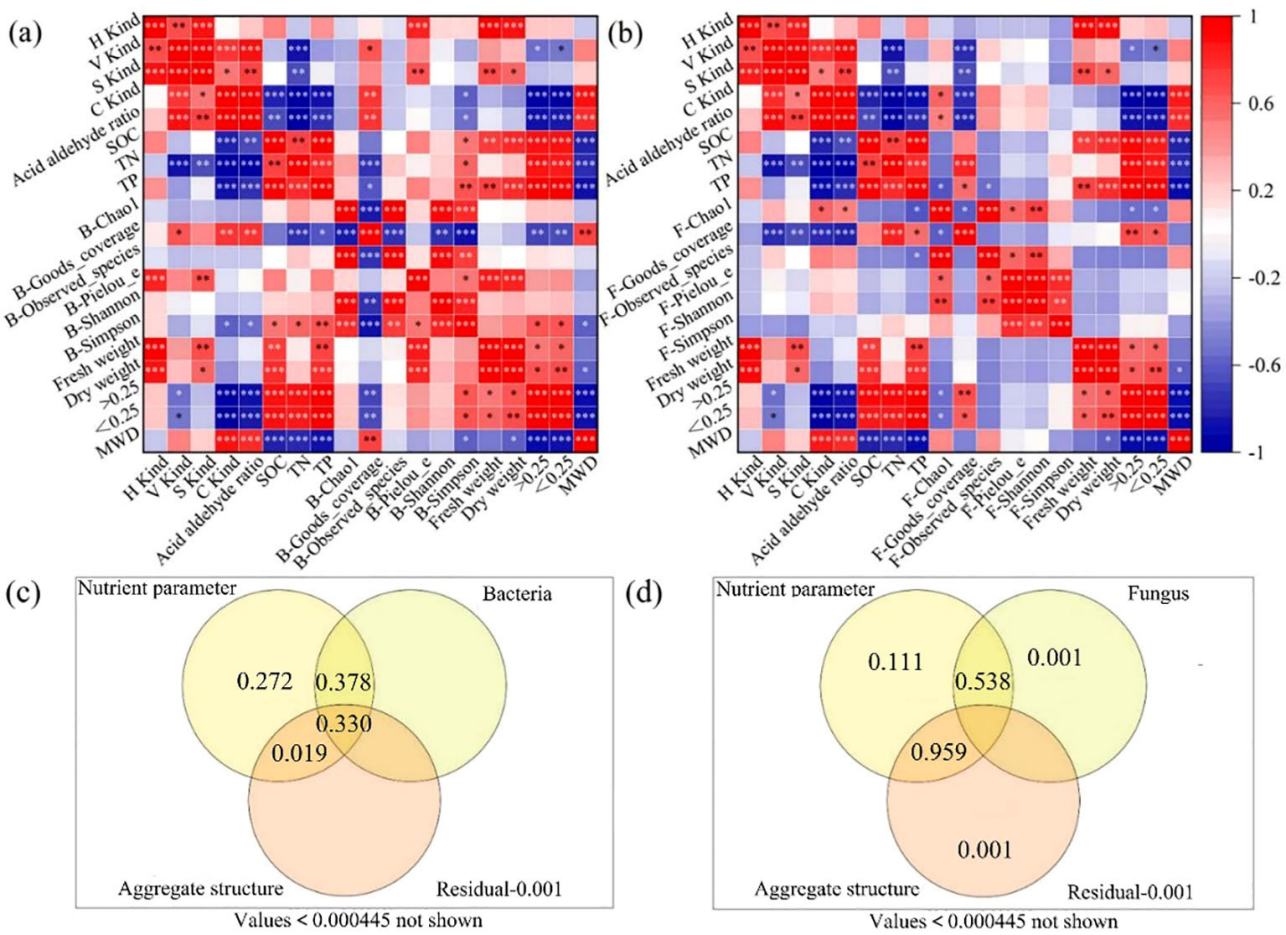
Correlation analysis and variance partitioning analysis (VPA) of soil physico-chemicals in successive years of leguminous green manure crop cultivation. **(a)** Correlation analysis of soil physico-chemicals in bacterial community-dominated system. **(b)** Correlation analysis of soil physico-chemicals in fungal community-dominated system. **(c)** VPA analysis of soil physico-chemicals on plant biomass in bacterial community-dominated system. **(d)** VPA analysis of soil physico-chemicals on plant biomass in fungal community-dominated system.

Correlation heatmaps (**Figures 5a, b**) revealed significant associations between bacterial and fungal communities and soil physicochemical properties as well as microbial diversity. In the bacterial community (**Figure 5a**), SOC was significantly positively correlated with bacterial diversity indices, including the Chao1 and Shannon indices, whereas the acid–formaldehyde ratio was negatively correlated with bacterial diversity. The influences of SOC and TN on the bacterial community were particularly pronounced, with strong correlations observed under the VVR treatment. In the fungal community (**Figure 5b**), both SOC and TN were significantly and positively correlated with fungal diversity metrics, such as the Simpson and Shannon indices.

The variance partitioning analysis (VPA) results demonstrated differential contributions of soil physicochemical properties, bacterial communities, and fungal communities to the variation in biomass. In the model incorporating the bacterial community (**Figure 5c**), the combined effect of soil chemical properties and the bacterial community accounted for the largest proportion of biomass variability, explaining 37.80% of the variation, followed by the joint influence of soil physical and chemical properties alongside the bacterial community, which explained 33.00%. In the fungal community model (**Figure 5d**), soil physicochemical properties alone contributed the most, accounting for 95.90% of the variation in biomass, followed by the combined effect of soil chemical properties and the fungal community, which explained 53.80%; the sole contribution of the fungal community was minimal at 0.10%.

## 4 Discussion

### 4.1 Relationships among soil physicochemical properties, microbial diversity and biomass

This study systematically elucidated the mechanistic pathway by which alfalfa cultivation enhances ecosystem functions in farmland reclaimed from coal mines. Specifically, the planting of alfalfa significantly increased the SOC and TP contents, enhanced microbial diversity, and subsequently promoted the accumulation of aboveground biomass, highlighting the synergistic contributions of soil physicochemical properties and microbial communities to ecological restoration. The observed increases in SOC and TP in the surface soil under alfalfa cultivation (**Table 1**) provided increased substrate availability and energetic influx for heterotrophic microbial metabolism for microbial communities (Zhang et al., 2024a). This enrichment not only directly stimulated microbial growth and activity but also indirectly modulated the composition and functional structure of the microbial community through alterations in the soil chemical environment.

Notably, alfalfa cultivation also affected the soil C:P and N:P iso-stoichiometric ratios, which were significantly greater than those in the control (**Table 1**). This shift may have alleviated phosphorus limitations and enhanced the efficiency of microbial acquisition of multiple elemental resources, thereby further contributing to increased microbial diversity. Specifically, microbial diversity indices—such as the bacterial Chao1 index, as well as the observed species and Shannon indices—were significantly increased in alfalfa-cultivated soils (**Table 4**), indicating that improved soil physicochemical properties positively influence microbial diversity (Wei et al., 2025b). The underlying mechanism may be attributed to the increase in soil organic carbon, which provides a broad range of carbon substrates, improves microhabitat conditions (e.g., aggregate stability and water retention), and reduces environmental stress, thereby fostering microbial diversity and establishing a positive feedback loop (Philippot et al., 2024).

Second, increased microbial diversity plays a pivotal role in biomass accumulation. Soil microorganisms contribute to increased fertility by decomposing organic matter and facilitating nutrient cycling, thereby creating favourable conditions for plant growth (Li et al., 2025b). In this study, the aboveground biomass in the alfalfa-cultivated plots was significantly greater than that in the other treatment plots (**Figure 2**), and this pattern was closely associated with the observed increase in microbial diversity. Enhanced microbial diversity not only improved the efficiency of nutrient turnover but also contributed to improved soil structure and functionality, ultimately fostering improved plant growth (Zhang et al., 2024b). This facilitative role of microbial communities is particularly critical in coal mine reclamation ecosystems, where microbial structure and function are often severely disturbed during the reclamation process (Li et al., 2024b).

In addition, a significant synergistic effect between soil physicochemical properties and microbial diversity was observed, which jointly influenced biomass accumulation. This synergy underscores the need for soil management and ecological restoration strategies to integrate both soil physicochemical conditions and microbial diversity to maximize ecosystem services and productivity (Hu et al., 2024). The VPA results confirmed that both soil physicochemical properties and microbial diversity contributed substantially to biomass variation (**Figures 5c**, **d**). The rhizosphere microbial community directly enhances the efficiency of plant nutrient acquisition through the combined effects of functional remodelling (e.g., nitrogen fixation, phytohormone synthesis) and increased microbial diversity, serving as the core biological driver of biomass accumulation. These findings emphasize the critical importance of maintaining microbial diversity in ecological restoration efforts (Guo et al., 2025; Chen et al., 2023).

### 4.2 Soil lignin drives soil organic carbon sequestration

This study demonstrated that alfalfa cultivation in soils reclaimed from coal mines significantly promoted the accumulation of lignin-derived compounds in the surface layer and may play a crucial role in stabilizing soil organic carbon and enhancing soil ecological functions. Specifically, the results showed that alfalfa planting markedly increased the abundance of lignin monomers, particularly in the 0–20 cm soil layer, where H-, V-, and S-type compounds clearly increased (**Figure 3**), thereby contributing to improvements in soil carbon pool stability and nutrient retention (Buse et al., 2024). Lignin, as a structurally complex and chemically recalcitrant macromolecule, is inherently resistant to microbial degradation, which prolongs its residence time in the soil matrix (Zhao et al., 2024). This biochemical persistence renders lignin a key factor in the long-term stabilization of soil organic matter and carbon sequestration. In this context, alfalfa cultivation significantly increased the accumulation of lignin-like compounds, which in turn promoted the potentially contributes to physical protection of soil organic matter through organ mineral associations and improved soil aggregation and structural stability (Li et al., 2024a). Moreover, the elevated acid–formaldehyde ratio observed under alfalfa treatment underscores the capacity of alfalfa to facilitate the transformation of organic matter into more stable humic fractions, highlighting the influence of plant-derived inputs on soil carbon fractionation pathways (Zhu et al., 2025). Therefore, lignin accumulation is likely to exert a regulatory effect on both the composition and metabolic functionality of soil microbial communities.

Lignin accumulation likely influences both the composition and metabolic potential of soil microbial communities. The decomposition of lignin predominantly depends on specialized microbial groups, particularly aromatic compound-degrading fungi such as white-rot fungi, which secrete an array of lignin-degrading enzymes (Zhang et al., 2021). These microbes are ecologically favoured in soils with elevated lignin contents, potentially enhancing the diversity and functional capabilities of the microbial community (Qiu et al., 2025). The lignin enrichment driven by alfalfa cultivation supplies these microbial taxa with a continuous and stable carbon substrate, thereby reinforcing soil biological activity and ecosystem resilience (Gao et al., 2025). Overall, alfalfa cultivation promotes the enrichment of lignin structural units, thereby enhancing not only the stability and structural maturity of soil organic matter but also the functional integrity of the soil system in reclaimed land by influencing microbial metabolic pathways. In summary, alfalfa planting supports both soil carbon sequestration and the restoration of microbial ecological functions in reclaimed soils through the accumulation of lignin and its derived structures.

### 4.3 The mechanism of soil quality improvement under green manure legume crop cultivation

This study contributes to elucidating the synergistic regulation of the physicochemical properties and aggregate structure of soil driven by green manure cultivation, which collectively contributes to soil quality improvement and biomass accumulation. A decade of continuous alfalfa cultivation markedly increased the organic carbon and total nitrogen contents in surface soils, and these changes were strongly correlated with increased plant biomass (**Figure 2** and **Table 1**), indicating that improved soil fertility provides a more favourable nutrient foundation for plant growth (Huang et al., 2025). Additionally, the enhancement of soil structure under alfalfa treatment was evidenced by increased aggregate stability (**Table 2** and **3**), a key factor in maintaining soil physical integrity, reducing erosion susceptibility, and promoting plant growth through improved water retention. The restoration capacity of legumes in mining ecosystems primarily stems from their biological nitrogen fixation ability and substantial inputs of organic matter (Zhou et al., 2025).

Alfalfa markedly increases soil nitrogen availability by converting atmospheric nitrogen into plant-accessible forms via symbiotic rhizobia (Liu et al., 2025). This nitrogen fixation process depends on the carbon substrates supplied by the host plant, which not only bolster plant growth but also improve soil structure and function through synergistic carbon–nitrogen interactions, thereby increasing soil water retention and nutrient supply efficiency (Wang et al., 2023). Furthermore, the increased stability of soil aggregates safeguards microbial habitats and protects soil organic carbon, contributing to increased functional redundancy and resilience within the soil ecosystem (Li et al., 2020).

In this context, the present study further substantiates the applicability and effectiveness of natural base solutions (NbS) in mine reclamation processes (Li et al., 2025b). The cultivation and management of leguminous green manure not only enhance soil quality but also facilitate the recovery of ecosystem services with reduced resource inputs, offering an economically and ecologically viable strategy for the management of degraded land (Yang et al., 2024; Wang et al., 2022a). The demonstrated success of the NbS approach highlights how plant–microbe interactions significantly improve the physicochemical properties and structure of soil, thereby creating conditions conducive to vegetation restoration and biomass accumulation on reclaimed surfaces (Wang et al., 2022b). Overall, the role of leguminous green manure in coal mine reclamation extends beyond soil quality and plant growth enhancement, providing critical support for improving soil system stability and sustainable utilization through aggregate structure improvements and the promotion of ecological processes. These findings further underscore the scientific validity and practical feasibility of employing legumes as pioneer species in land degradation management and establish a replicable ecological restoration framework for future reclamation efforts (Liu et al., 2024).

This study highlights the restorative effects of legumes in coal mine reclamation; however, several limitations remain. First, although this study was based on a long-term field trial of coal mine reclamation, the generalizability of these findings across different climatic zones, soil types, and mining conditions requires validation. Additionally, while this study controlled for soil spatial heterogeneity, its precise influence on reclamation outcomes and microbial community responses has yet to be systematically quantified. Future research should be expanded to multiregional trials to compare the reclamation potential of diverse legume species and integrate multitopic approaches to comprehensively characterize microbial functional genes and their expression patterns. Such advances will enhance the development of tailored strategies for ecological restoration in mining-impacted areas.

## 5 Conclusions

Based on long-term field experiments, this study comprehensively evaluated the role of green manure legumes in the restoration of soil quality within coal mine reclamation areas from multiple perspectives, including soil physicochemical properties, microbial community structure, lignin content, and plant biomass. The results demonstrated that leguminous green manure cultivation significantly increased the soil total nitrogen and total phosphorus contents, improved the soil structure and aggregate formation, and enhanced the overall soil physicochemical characteristics. Furthermore, the synergistic interaction between lignin accumulation and microbial community activation as a fundamental mechanism for stable carbon sequestration. By integrating these functional pathways into the core strategic framework of land remediation, this study highlights the potential of legumes as a sustainable Nature-based Solution (NbS) for the long-term ecological restoration of degraded mining sites worldwide. This study highlights legumes, as representative green manure species, have markedly facilitated the comprehensive recovery of soil ecological functions in coal mine reclamation areas through multiple ecological pathways, including nitrogen fixation, nutrient replenishment, structural improvement, microbial activation, carbon sequestration, and biomass enhancement. In summary, the importance of integrating green manure plants and their functional mechanisms into the core strategic framework of future mine reclamation and land remediation practices to achieve the systematic, long-term, and sustainable ecological restoration of degraded lands.

## Data Availability

The datasets presented in this study can be found in online repositories. The names of the repository/repositories and accession number(s) can be found below: https://data.mendeley.com/drafts/z77d4bh34h.

## References

[B1] Abiven S. Heim A. Schmidt M. W. I. (2011). Lignin content and chemical characteristics in maize and wheat vary between plant organs and growth stages: consequences for assessing lignin dynamics in soil. Plant Soil 343, 369–378. doi: 10.1007/s11104-011-0725-y 30311153

[B2] Ahsan T. Tian P. C. Gao J. Wang C. Liu C. Huang Y. Q. (2024). Effects of microbial agent and microbial fertilizer input on soil microbial community structure and diversity in a peanut continuous cropping system. J. Adv. Res. 64, 1–13. doi: 10.1016/J.JARE.2023.11.028 38030126 PMC11464484

[B3] Akchaya K. Parasuraman P. Pandian K. Vijayakumar S. Thirukumaran K. Mustaffa M. R. A. F. . (2025). Boosting resource use efficiency, soil fertility, food security, ecosystem services, and climate resilience with legume intercrop: a review. Front. Sustain. Food. Syst. 9, 1527256. doi: 10.3389/FSUFS.2025.1527256

[B4] Bao S. D. (2000). Soil Agrochemical Analysis (Beijing: China Agricultural Publishing House), 25–106.

[B5] Barbieri P. Starck T. Voisin A. S. Nesme T. (2023). Biological nitrogen fixation of legumes crops under organic farming as driven by crop management: a review. Agric. Syst. 205, 103579. doi: 10.1016/j.agsy.2022.103579 38826717

[B6] Bianchi T. S. Mayer L. M. Amaral J. H. Arndt S. Galy V. Kemp D. B. . (2024). Anthropogenic impacts on mud and organic carbon cycling. Nat. Geosci. 17, 287–297. doi: 10.1038/S41561-024-01405-5 37880705

[B7] Buse K. K. Carroll A. L. Bradford B. J. Min D. Jagadish K. Kononoff P. J. (2024). The effect of replacing conventional alfalfa hay with lower-lignin alfalfa hay on feed intake, nutrient digestibility, and energy utilization in lactating Jersey cows. J. Dairy Sci. 107, 9379–9389. doi: 10.3168/JDS.2024-24966 39067760

[B8] Chen S. Wang Y. Gao J. Chen X. Qi J. Peng Z. . (2023). Agricultural tillage practice and rhizosphere selection interactively drive the improvement of soybean plant biomass. Plant Cell Environ. 46, 3542–3557. doi: 10.1111/PCE.14694 37564021

[B9] Gao Q. Wang L. Fang Y. Gao Y. Ma L. Wang X. . (2025). Conservation agriculture boosts topsoil organic matter by restoring free lipids and lignin phenols biomarkers in distinct fractions. Soil Tillage Res. 248, 106463. doi: 10.1016/J.STILL.2025.106463 38826717

[B10] Griffin A. J. Jungers J. M. (2025). Effects of intercrop perennial legumes on intermediate wheatgrass productivity. Field Crops Res. 330, 109954. doi: 10.1016/J.FCR.2025.109954 38826717

[B11] Guan Y. Wang J. Zhou W. Bai Z. Cao Y. (2022). Identification of land reclamation stages based on succession characteristics of rehabilitated vegetation in the Shuo opencast coal mine. J. Environ. Manage. 305, 114352. doi: 10.1016/J.JENVMAN.2021.114352 34973560

[B12] Guo H. Guan M. Wang Y. Li X. Wang H. He C. . (2025). Research on the synergistic optimization of Scutellaria baicalensis yield, quality and soil environment by key microorganisms, driven by the combined approach of reducing chemical fertilizer usage while incorporating organic fertilizer. Ind. Crops Prod. 229, 121044. doi: 10.1016/J.INDCROP.2025.121044 38826717

[B13] Hao S. Hei Z. Ma J. Shao Q. Cai T. Hu H. W. . (2024). Soil microbial diversity-function relationships are changed by human activity at a landscape scale. Plant Soil 513, 137–151. doi: 10.1007/S11104-024-07174-9 30311153

[B14] Hu Q. Zhang Y. Cao W. Yang Y. Hu Y. He T. . (2024). Legume cover crops sequester more soil organic carbon than non-legume cover crops by stimulating microbial transformations. Geoderma 450, 117024. doi: 10.1016/j.geoderma.2024.117024 38826717

[B15] Huang H. Zhang Z. Wu Q. Liu Z. Wang Q. Ying Y. . (2025). Global comprehensive evaluation shows that green manure enhances crop productivity while mitigating gaseous nitrogen losses. Resour. Conserv. Recycl. 220, 108351. doi: 10.1016/J.RESCONREC.2025.108351 38826717

[B16] Huang N. He H. Y. Fan R. Li X. Y. Zhao C. M. Li J. H. (2025). Planting of nitrogen-fixing shrubs promotes soil carbon sequestration by increasing mineral-associated organic fraction. Geoderma 457, 117282. doi: 10.1016/J.GEODERMA.2025.117282 38826717

[B17] Kanté M. Riah-Anglet W. Cliquet J. B. Trinsoutrot-Gattin I. (2021). Soil enzyme activity and stoichiometry: linking soil microorganism resource requirement and legume carbon rhizodeposition. Agronomy 11, 2131. doi: 10.3390/AGRONOMY11112131 30654563

[B19] Li J. Dong L. Fan M. Shangguan Z. (2024a). Long-term vegetation restoration promotes lignin phenol preservation and microbial anabolism in forest plantations: implications for soil organic carbon dynamics. Sci. Total Environ. 928, 172635. doi: 10.1016/J.SCITOTENV.2024.172635 38643876

[B21] Li M. He M. Lu Y. Lu W. Wang P. Zhang Y. . (2025a). Synergistic benefits of leguminous green manure intercrop for weed control and productivity improvement in pear orchards. Sci. Hortic. 340, 113955. doi: 10.1016/j.scienta.2025.113955 38826717

[B18] Li D. Li S. Chen H. Wu J. (2025b). Reseeding promotes plant biomass by improving microbial community stability and soil fertility in a degraded subalpine grassland. Geoderma 453, 117160. doi: 10.1016/J.GEODERMA.2024.117160 38826717

[B22] Li Z. Li J. Li T. Zhang Q. Gao C. Lu J. . (2024b). Effects of functional microbial agents on the microbial community and fertility of reclaimed soil in a coal mining area. Environ. Technol. Innov. 36, 103891. doi: 10.1016/J.ETI.2024.103891 38826717

[B20] Li J. Yuan X. Ge L. Li Q. Li Z. Wang L. . (2020). Rhizosphere effects promote soil aggregate stability and associated organic carbon sequestration in rocky areas of desertification. Agric. Ecosyst. Environ. 304, 107126. doi: 10.1016/j.agee.2020.107126 38826717

[B23] Lira Junior M. A. Fracetto F. J. C. Ferreira J. D. S. Silva M. B. Fracetto G. G. M. (2020). Legume silvopastoral systems enhance soil organic matter quality in a subhumid tropical environment. Soil Sci. Soc Am. J. 84, 1209–1218. doi: 10.1002/saj2.20106 41531421

[B24] Liu R. Li C. Zhang Y. Liu C. Xue J. Zheng Y. . (2025). Enhanced biological nitrogen fixation and nodulation in alfalfa through the synergistic interactions between Sinorhizobium meliloti and Priestia aryabhattai. World J. Microbiol. Biotechnol. 41, 1–14. doi: 10.1007/S11274-025-04394-8 40415123

[B25] Liu Z. Liu S. Gao L. Li J. Li X. Gao Z. . (2024). Long-term recovery of compacted reclaimed farmland soil in coal mining subsidence area. Ecol. Indic. 168, 112758. doi: 10.1016/J.ECOLIND.2024.112758 38826717

[B27] Ma X. Qu H. Liao S. Ji Y. Li J. Chao L. . (2023). Changes in assembly processes and differential responses of soil microbial communities during mining disturbance in mining reclamation and surrounding grassland. Front. Microbiol. 14, 1230817. doi: 10.1016/j.catena.2023.107332. Catena, 231, 107332. 38826717

[B28] Ma Y. Xiao C. Liu J. Ren G. (2025). Nutrient-dependent regulation of symbiotic nitrogen fixation in legumes. Hortic. Res. 12, uhae321. doi: 10.1093/HR/UHAE321 40046318 PMC11879230

[B26] Ma T. Zhu S. Wang Z. Chen D. Dai G. Feng B. . (2018). Divergent accumulation of microbial necromass and plant lignin components in grassland soils. Nat. Commun. 9, 3480. doi: 10.1038/s41467-018-05891-1 30154479 PMC6113315

[B29] Mao X. Sun T. Zhu L. Wanek W. Cheng Q. Wang X. . (2024). Microbial adaption to stoichiometric imbalances regulated the size of soil mineral-associated organic carbon pool under continuous organic amendments. Geoderma 445, 116883. doi: 10.1016/J.GEODERMA.2024.116883 38826717

[B30] Philippot L. Chenu C. Kappler A. Rillig M. C. Fierer N. (2024). The interplay between microbial communities and soil properties. Nat. Rev. Microbiol. 22, 226–239. doi: 10.1038/s41579-023-00980-5 37863969

[B31] Qiu Q. Sun X. Li H. Zhang F. Zhou D. Tian K. . (2025). Biodegradation of polystyrene and its mechanisms driven by a customized lignin-degrading microbial consortium and degrading bacteria. J. Environ. Manage. 384, 125560. doi: 10.1016/j.jenvman.2025.125560 40311357

[B32] Ren Q. Liu G. Liu C. Qiang F. Ai N. (2023). Soil quality evaluation and driving factor analysis of Hippophae rhamnoides plantations in coal mine reclamation areas based on different restoration durations. Forests 14, 1425. doi: 10.3390/F14071425 30654563

[B33] Ren Q. Qiang F. Liu G. Liu C. Ai N. (2025). Response of soil quality to ecosystems after revegetation in a coal mine reclamation area. Catena 257, 109038. doi: 10.1016/j.catena.2025.109038 38826717

[B34] Shao Y. Xu Q. Wei X. (2023). Progress of mine land reclamation and ecological restoration research based on bibliometric analysis. Sustainability 15, 10458. doi: 10.3390/SU151310458 30654563

[B35] Stainsby A. May W. E. Lafond G. P. Entz M. H. (2020). Soil aggregate stability increased with a self-regenerating legume cover crop in low-nitrogen, no-till agroecosystems of Saskatchewan, Canada. Can. J. Soil Sci. 100, 314–318. doi: 10.1139/cjss-2019-0110 38826717

[B36] Wang W. Dong Y. Wang H. B. Xue D. H. Zhang H. F. Li Z. J. . (2025). Long-term legume cultivation affects the soil bacterial community via altering the soil pore structure in coal mine reclamation agroecosystems. Plant Cell Environ. 49 (2), 412–427. doi: 10.1111/pce.70063 40653865

[B37] Wang W. Wang B. Z. Zhou R. Ullah A. Zhao Z. Y. Wang P. Y. . (2022a). Biocrusts as a nature-based strategy (NbS) improve soil carbon and nitrogen stocks and maize productivity in semiarid environment. Agric. Water Manage. 270, 107742. doi: 10.1016/J.AGWAT.2022.107742 38826717

[B39] Wang Y. Wu P. Qiao Y. Li Y. Liu S. Gao C. . (2023). The potential for soil C sequestration and N fixation under different planting patterns depends on the carbon and nitrogen content and stability of soil aggregates. Sci. Total Environ. 897, 165430. doi: 10.1016/J.SCITOTENV.2023.165430 37437631

[B38] Wang W. Zhou R. Wang B. Z. Zhao L. Zhao Z. Y. Sheteiwy M. S. . (2022b). Biocrust as a nature-based strategy (NbS) to restore the functionality of degraded soils in semiarid rainfed alfalfa (Medicago sativa L.) field. J. Clean. Prod. 336, 130378. doi: 10.1016/j.jclepro.2022.130378 38826717

[B40] Wei C. Cao B. Gao S. Liang H. (2025a). Co-incorporation of green manure and rice straw increases rice yield and nutrient utilization. Plants 14, 1678. doi: 10.3390/PLANTS14111678 40508350 PMC12157964

[B41] Wei K. Sun Y. Cartmill A. D. López I. F. Ma C. Zhang Q. (2025b). Long-term effects of nitrogen and phosphorus fertilizers on rhizosphere physicochemical characteristics and microbial composition in alfalfa. Ind. Crops Prod. 227, 120776. doi: 10.1016/J.INDCROP.2025.120776 38826717

[B42] Wu Y. Sun Z. Liu R. Wang L. Cai B. (2024). Enhancing sulfur absorption in soybean rhizosphere through arbuscular mycorrhizal fungi inoculation: implications for soil health and crop growth. J. Clean. Prod. 463, 142759. doi: 10.1016/J.JCLEPRO.2024.142759 38826717

[B43] Yang W. Qu G. Kelly A. R. Wu G. L. Zhao J. (2024). Positive effects of leguminous shrub encroachment on multiple ecosystem functions of alpine meadows and steppes greatly depended on increasing soil nutrient. Catena 236, 107745. doi: 10.1016/j.catena.2023.107745 38826717

[B44] Yasen M. Li M. Wang J. (2025). The diversity pattern of soil bacteria in the rhizosphere of different plants in mountain ecosystems. World J. Microbiol. Biotechnol. 41, 1–12. doi: 10.1007/S11274-025-04299-6 40011276

[B45] Zhang B. Hu X. Zhao D. Wang Y. Qu J. Tao Y. . (2024a). Harnessing microbial biofilms in soil ecosystems: enhancing nutrient cycling, stress resilience, and sustainable agriculture. J. Environ. Manage. 370, 122973. doi: 10.1016/J.JENVMAN.2024.122973 39437688

[B46] Zhang W. Wang W. Wang J. Shen G. Yuan Y. Yan L. . (2021). Isolation and characterization of a novel laccase for lignin degradation, LacZ1. Appl. Environ. Microbiol. 87, e01355–e01321. doi: 10.1128/AEM.01355-21 34524901 PMC8580002

[B47] Zhang X. Zhang F. Yuan Z. Q. Li F. M. (2024b). Alfalfa-livestock system promotes the accumulation of soil organic carbon in a semi-arid marginal land. Agric. Ecosyst. Environ. 375, 109200. doi: 10.1016/J.AGEE.2024.109200 38826717

[B48] Zhao B. Dong W. Chen Z. Zhao X. Cai Z. Feng J. . (2024). Microbial inoculation accelerates rice straw decomposition by reshaping structure and function of lignocellulose-degrading microbial consortia in paddy fields. Bioresour. Technol. 413, 131545. doi: 10.1016/j.biortech.2024.131545 39341423

[B49] Zhou G. Li G. Liang H. Liu R. Ma Z. Gao S. . (2025). Green manure coupled with straw returning increases soil organic carbon via decreased priming effect and enhanced microbial carbon pump. Glob. Change Biol. 31, e70232. doi: 10.1111/gcb.70232 40329747

[B50] Zhu D. Sun L. Mao L. Li J. Yan B. Li B. . (2025). Combined effects of crop alfalfa (Medicago sativa L.) on the soil pore structure, microbial communities and organic carbon fractions in saline soils. Appl. Soil Ecol. 208, 105993. doi: 10.1016/j.apsoil.2025.105993 38826717

